# Postoperative incision scars and cosmetic satisfaction of living liver donors

**DOI:** 10.1097/MD.0000000000026187

**Published:** 2021-06-11

**Authors:** Ya-Lan Hsu, Chia-En Hsieh, Ping-Yi Lin, Shin-Lung Lin, Kuo-Hua Lin, Li-Chueh Weng, Yao-Li Chen

**Affiliations:** a Nurse Practitioner of liver transplantation, Department of Nursing, Changhua Christian Hospital, Changhua; b Department of Nursing, Hung Kung University, Taichung; c Department of Plastic and Reconstruction Surgery; d Department of General Surgery, Changhua Christian Hospital, Changhua; e Associate Professor, Department of Nursing, Graduate Institute of Clinical Medical Science, Chang Gung University, Taoyuan, Taiwan.

**Keywords:** cosmetic satisfaction, living liver donor, postoperative incision scar

## Abstract

Cosmetic appearance is a major concern for living donors. However, little is known about the impact of a surgical scar on body image changes in living liver donors. The aim of this study was to identify potential factors that cause displeasing upper midline incision scar, and to evaluate the overall satisfaction regarding body image and scarring after living donor hepatectomy.

Donors who underwent right lobe hepatectomy were recruited. Exclusion criteria included reoperation, refusal to participate, and lost follow-up. All donors were invited to complete the Vancouver Scar Scale (VSS) and the body image questionnaire. According to the VSS results of upper midline incision scar, donors were divided into 2 groups: good scarring group (VSS ≤4) and bad scarring group (VSS >4). we compared the clinical outcomes, including the demographics, preoperation, intraoperation, and postoperation variables. The study also analyzed the results of the body image questionnaire.

The proportion of male donors was 48.9%. The bad scarring group consisted of 63% of the donors. On multivariate analysis, being a male donor was found to be an independent predictor of a cosmetically displeasing upper midline incision scar with statistical significance. The results of body image questionnaires, there were significant differences in cosmetic score and confidence score among the 2 groups.

The upper midline incision and male donors have higher rates of scarring in comparison with the transverse incision and female donors. Donors who reported having a higher satisfaction with their scar appearance usually had more self-confidence. However, the body image won’t be affected. Medical staff should encourage donors to take active participation in wound care and continuously observe the impact of surgical scars on psychological changes in living liver donors.

## 1 Introduction

There has been an extreme shortage of cadaveric donors in Asian countries, so living donors constitute the majority of organ sources for liver transplantations in Asia. Living donor liver transplantation has been an effective alternative treatment for end-stage liver disease.^[1]^ However, an abdominal incision is inevitable in the living donor as in other patients. An inverted L incision was the most common approach, and it has been used for many years. This conventional incision allows for sufficient exposure of all segments of the liver in the limited visualization of the surgical field and complete access for dissecting the inferior vena cava and right hepatic vein, as well as the parenchymal separation.^[2]^ One study determined that the transverse incision scar was found to be significantly shorter and narrower than the midline incision scar.^[3]^ Scars often take six months to 1 year to complete and mature.^[4–6]^

Collagen is synthesized, degraded, reorganized, and stabilized via molecular crosslinking in the remodeling phase; this leads to the final result of microscopic and macroscopic scar formation. If more collagen is deposited, a hypertrophic scar will take shape.^[7]^ Research shows scarring may cause patient discomfort and psychological stress.^[8]^ However, few studies in the living donor literature have investigated the psychological impact of scars on donors. The purpose of this study was to determine the independent risk factors for poor upper midline incision scarring and to inspect the postdonation levels of body image and scar satisfaction according to the scarring status.

## 2 Materials and methods

### 2.1 Participants

**Figure 1:**
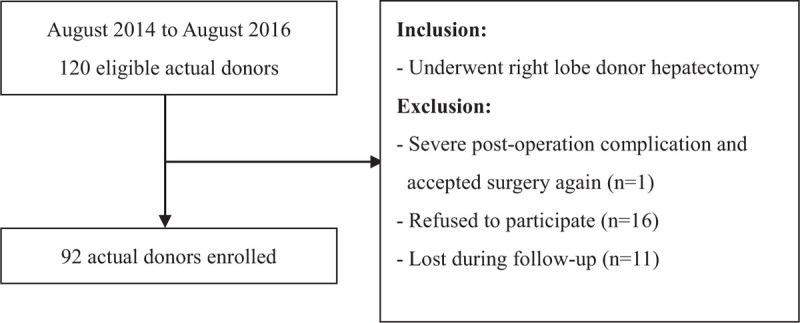
Flow chart of participant's inclusion and exclusion.

This cross-sectional study was approved by the institutional review board of Changhua Christian Hospital (181219). Living donors who were scheduled to undergo right lobe donor hepatectomy at our hospital during the period of August 2014 to August 2016 were recruited. At the time of enrollment, participants were at least 1-year postdonation. Postoperative scar care protocol was combining Steri-Strips and Dermatix Ultra. The protocol was carried out 1-month postdonation and continued for 6 months. All participants voluntarily joined the study and gave written informed consent. The inclusion criterion was having undergone right lobe donor hepatectomy. The exclusion criteria included severe postoperation complications and acceptance of surgery again, refusal to participate, and loss during follow-up for any reason. During the period of study recruitment, 120 living donors were eligible. Of these, 92 participated, 1 reoperation, 16 refused to participate, and 11 were lost during follow-up (Fig. 1).

### 2.2 Vancouver scar scale

The Vancouver scar scale (VSS) rated four physical characteristics of scars: vascularity, pigmentation, pliability, and height.^[9,10]^ The total score (ranging from 0–13) was obtained by summing the scores of the four parameters. The lower the score, the more the scar represented normal skin. Other studies have provided evidence that the VSS is a reliable and effective device to assess linear surgical scars.^[11,12]^ Internal consistency was acceptable for the VSS, with Cronbach alpha values ranging from 0.71∼0.79.^[11,12]^ For these reasons, the study used the VSS as the standardized scar assessment tool. Two occupational nurse practitioners with more than 6 years of experience each in liver transplant units assessed the physical characteristics of the scars. They accepted training from a plastic surgeon. The training included a 30-minute session in which observers were taught in detail the four physical characteristics of scars used in the VSS. The study proceeded with the assessment of three donors’ scars; the interobserver reliability was excellent for the VSS score (0.805). A scar was classified as a poor scar if its total VSS score was higher than 4.

### 2.3 Body image questionnaire (BIQ)

The body image questionnaire (BIQ) was previously described and applied by Dunker in 1998.^[13]^ Donors completed the BIQ, which has been demonstrated on factor analysis to produce two scales: the body image scale (BIS) and the cosmetic scale (CS). The BIS (items 1–5) investigates the patient's perception of and satisfaction with their own body and assesses the patient's attitudes toward their bodily appearance. It has five questions answered on a Likert scale ranging from 1 (no, not at all) to 4 (yes, extremely). The total BIS has a minimum of 5 and a maximum of 20. The CS (items 6–8) evaluates the donor's satisfaction with the physical appearance of their scar. The CS has 3 questions answered on a Likert scale: 2 questions ranging from 1 (very unsatisfied) to 7 (very satisfied) and a third question ranging from 1 (very unsatisfied) to 10 (very satisfied). The total CS score has a minimum of 3 and a maximum of 24. Note that on the BIQ a higher score indicates greater satisfaction on both scales. The reliability coefficients (values of Cronbach alpha) for BIS and CS were 0.81 and 0.74, respectively.^[14]^ Two items (9, 10) about the patient's self-confidence before and after surgery have been added to the final version of the BIQ; they are answered on a Likert scale ranging from 1 (not confident) to 10 (very confident). Predonation confidence ratings were subtracted from postdonation confidence ratings to determine the donors who felt their confidence decreased after donation. This questionnaire has been used and tested in two studies involving living donor nephrectomy and living donor hepatectomy.^[2,4]^

### 2.4 Assessment

During the follow-ups in the outpatient clinic, the donors were invited to complete the VSS and BIQ. One donor will have 2 VSS, one is transverse incision scar's VSS and the other is upper midline incision scar's VSS. According to the upper midline incision scar assessment results, all donors were divided into two groups: VSS **≤ **4 (good scarring group, n = 34, 37%) and VSS **> **4 (bad scarring group, n = 58, 63%). The two groups were compared based on demographics and preoperation, intraoperation and postoperation related variables. The following demographic data were collected: gender, age, and body mass index (BMI). Preoperative blood examination data included white blood cells, C-reactive protein, direct bilirubin, total bilirubin, aspartate aminotransferase, alanine aminotransferase, and γ-glutamyltransferase. Intraoperative variables included operation time, blood loss, residual liver volume and also the percentage of fatty change (<10% or ≥10%), which was determined from a liver biopsy. Postoperative variables included complications and hospital days.

### 2.5 Surgical techniques

All donors were administered endotracheal anesthesia in the supine position. A central venous catheter was used to monitor the hemodynamics. An inverted L incision was made from the xiphoid process to the umbilicus incision on the sagittal plane and was extended to the right rectus abdominis muscle on the transverse plane. Incision length was dependent on BMI, the graft volume, and the shape of the abdominal cavity. The vast majority of the upper midline incisions ranged from 8 to 10 cm in length, and the transverse incisions ranged from 10 to 12 cm and were made in the right lower quadrant area. The percentage of liver volume donated depended on what size could provide adequate graft volume to the recipient while leaving sufficient residual liver in the donor to ensure the donor's safety. The surgeons needed to mobilize the liver and dissect the hepatic hilum and parenchyma. A hepatic parenchymal transection was carried out using a cavitational ultrasonic surgical aspirator. After the bile duct was divided, the graft was removed. After the completion of hemostasis, a closed suction drain was placed into the right subphrenic space to observe any signs of bleeding. The abdominal incision is closed layer by layer. The other general surgery surgeon was responsible for abdominal fascia suture and the plastic surgeon was responsible for subepidermal suture. The suture process was by the same general surgery surgeon and plastic surgeon. First, the abdominal muscle fascial layers (anterior and posterior rectus fascia) are reapproximated by continuous running suture with monofilament synthetic absorbable suture, polydioxanone (PDS Plus suture 1, PDP9237T, MO-2 150 cm, Ethicon, Somerville, NJ). Then, the Scarpa’ fascia is closed by interrupted suture with PDS plus Suture 4-0 (PDP496, PS-2 45 cm, Ethicon, Somerville, NJ). Finally, subcutaneous closure is finished by continuous running suture with PDS plus Suture 4-0 (PDP496, PS-2 45 cm, Ethicon, Somerville, NJ).

### 2.6 Statistical analysis

Demographic characteristics were analyzed using descriptive statistics including ranges, means, standard deviations, and proportions as appropriate. The paired-samples *t* test compared 2 VSS scores (transverse incision and upper midline incision) that were from the same donor. For intergroup comparisons, the normal distribution of data was first evaluated with the Kolmogorov–Smirnov test. Descriptive variables were analyzed using the chi-square test or Fisher exact test. The groups were compared with the independent two-sample *t* test or Wilcoxon rank-sum test. Univariate and multivariate analyses of data from all patients were performed. The Pearson product-moment correlation coefficient was used to measure the strength of a linear association between VSS and BIQ. Statistical significance was confirmed when a *P* value was < .05. All analyses were conducted with SPSS 20.0 (SPSS Inc, Chicago, IL).

## 3 Results

### 3.1 Sample details

Detailed characteristics of the 92 donors are presented in Table 1. The proportion of male donors was 48.9%. The mean age of the donors was 34.96 ± 8.76 years. The mean BMI was 23.20 ± 3.39 (kg/m^2^). The pre-operative blood examination results, including white blood cells, C-reactive protein, direct bilirubin, total bilirubin, aspartate aminotransferase, alanine aminotransferase and γ-glutamyltransferase, were all in acceptable ranges. The mean operation time was 232.55 ± 49.81 minutes and the mean blood loss was 174.78 ± 112.55 ml. The mean residual liver volume was 586.77 ± 202.55 ml. In addition, 20.7% of the donors presented fatty change in the liver. There was no donor mortality, but postoperative complications were observed in 43.5% of the donors. The patients were hospitalized for 9.27 ± 2.01 days after surgery.

**Table 1:** Demographic and Clinical Characteristics for 92 living donors.

Characteristic (n = 92)	Data
Male sex	45 (48.9%)
Age	34.96 ± 8.76 (21-54)
BMI (kg/m^2^)	23.20 ± 3.39 (17.4–30.8)
Preoperative blood examination	
WBC (∗10^3^/μL)	6.72 ± 1.55 (3.1–10.4)
CRP (mg/dL)	0.12 ± 0.19 (0.02–1.36)
Bil-D (mg/dL)	0.13 ± 0.04 (0.10–0.30)
Bil-T (mg/dL)	0.70 ± 0.27 (0.19–1.55)
AST (U/L)	22.17 ± 5.21 (14–41)
ALT (U/L)	20.07 ± 10.76 (6–56)
γ-GT (U/L)	20.76 ± 24.68 (6–135)
Intraoperative	
Operation time (min)	232.55 ± 49.81 (155–505)
Blood loss (ml)	174.78 ± 112.55 (30–600)
Fatty liver	19 (20.7%)
Residual liver volume (ml)	586.77 ± 202.55 (254.66–1669.15)
Postoperative	
Complication	40 (43.5%)
Hospital days	9.27 ± 2.01 (7–20)

γ-GT = γ-glutamyltransferase, ALT = alanine aminotransferase, AST = aspartate aminotransferase, Bil-D = direct bilirubin, Bil-T = total bilirubin, BMI = body mass index, CRP = C-reactive protein, WBC = white blood cell.

### 3.2 VSS after donor hepatectomy

**Figure 2:**
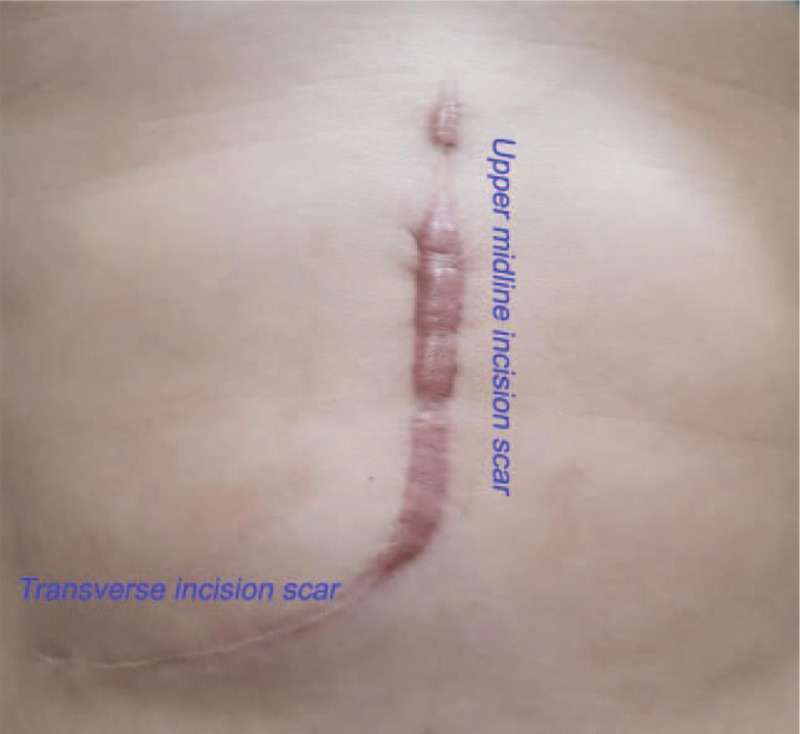
The study evaluated the different parts of the same incision scar in the living donor. The upper midline incision usually extends from the xiphoid process to the umbilicus. The transverse incision was made in the right lower quadrant of the abdomen.

The study used VSS to investigate the different parts of the same incision scar: transverse incision scars and upper midline incision scar in one donor (Fig. 2). The result as following: the mean donor's VSS was 3.78 ± 2.89 (range 0–10) for transverse incision scars and 5.75 ± 3.34 (range 0–11) for upper midline incision scars. The upper midline incision scars were obviously worse than the transverse incision scars (*P* < .001).

### 3.3 Risk factors for upper midline incision scar

All participants were divided into 2 groups to compare the demographics and preoperation, intraoperation and postoperation variables. The risk factors associated with upper midline incision scars in the living donors are listed in Table 2. The following characteristics were associated with significantly increased risks of poor scar healing: male donors (*P* = .004) and preoperative high BMI (*P* = .015). On univariate analysis, male donors (dds ratio [OR] = 3.652, 95% CI = 1.476–9.038, *P* = .005) and preoperative high BMI (OR = 1.174, 95% CI = 1.023–1.348, *P* = .022) were significant factors. On multivariate analysis, being a male donor was found to be an independent predictor of a cosmetically displeasing upper midline incision scar with statistical significance (OR = 2.825, 95% CI = 1.052–7.589, *P* = .039) (Table 3).

**Table 2:** Risk factors analysis for upper midline incision scar in the living donors.

Variable	Good scarring group (n = 34)	Bad scarring group (n = 58)	*P*
	n (%)	n (%)	
Gender			.004
Male	10 (29.4%)	35 (60.3%)	
Female	24 (70.6%)	23 (39.7%)	
Fatty liver			.107
No	30 (88.2%)	43 (74.1%)	
Yes	4 (11.8%)	15 (25.9%)	
Postoperation complication			.733
No	20 (58.8%)	32 (55.2%)	
Yes	14 (41.2%)	26 (44.8%)	
	Mean ± SD (Range)	Mean ± SD (Range)	
Age	34.35 ± 8.27 (21–49)	35.31 ± 9.09 (22–54)	.616
Preoperative			
BMI (kg/m^2^)	22.13 ± 2.99 (17.4–30.4)	23.84 ± 3.49 (17.7–30.8)	.015
WBC(∗10^3^/μL)	6.59 ± 1.71 (3.1–10.4)	6.81 ± 1.462 (3.8–9.8)	.514
CRP(mg/dL)	0.09 ± 0.10 (0.02–0.40)	0.15 ± 0.23 (0.02–1.36)	.151
Intraoperative			
Operation time (min)	232.35 ± 58.63 (165–505)	232.67 ± 44.40 (155–415)	.977
Blood loss (ml)	175.00 ± 97.12 (50–400)	174.66 ± 121.51 (30–600)	.989

BMI = body mass index, CRP = C-reactive protein, SD = standard deviations, WBC = white blood cell.

**Table 3:** Univariate and multivariate analyses for upper midline incision scar in the living donors.

	Univariate analysis			Multivariate analysis		
	(Good scarring vs bad scarring)			(Good scarring vs bad scarring)		
Variable	Odds ratio	95% CI	*P*	Odds ratio	95% CI	*P*
Gender (male)	3.652	1.476–9.038	.005	2.825	1.052–7.589	.039
Fatty Liver (No fatty)	0.382	0.115–1.266	.382			
Postoperation complication (No)	1.161	0.493–2.734	.733			
Age	1.013	0.964–1.603	.611			
Preoperative						
BMI (kg/m^2^)	1.174	1.023–1.348	.022	1.099	1.052–7.589	.207
WBC (∗10^3^/μL)	1.097	0.833–1.445	.510			
CRP (mg/dL)	10.944	0.343–349.702	.176			
Intraoperative						
Operation time (min)	1.000	0.992–1.009	.976			
Blood loss (ml)	1.000	0.996–1.004	.989			

BMI = body mass index, CRP = C-reactive protein, WBC = white blood cell.

### 3.4 Body image and cosmetic satisfaction after donor hepatectomy

The results of the body image questionnaires for the good scarring group and bad scarring group were summarized in Table 4. The good scarring group had higher average scores across all questions. There were no significant differences in BIS between the two groups (*P* = .057). The total CS score in the good scarring group was 18.88 ± 4.89, and it was 14.48 ± 4.48 in the bad scarring group (*P* < .001). The total self-confidence score in the good scarring group was 15.71 ± 3.04, and it was 14.38 ± 3.071 in the bad scarring group (*P* = .048). The significant differences between the two groups in CS score and self-confidence score were noted. Living donors who presented lower cosmetic satisfaction and self-confidence did so because of more obvious scar formation. Pearson's product-moment correlation was run to assess the relationship between the VSS and 3 subscales of BIQ (Table 5). There was moderate negative correlation between the VSS and CS, *r* = −0.464, *P* < .01. This means that when scarring was more obvious, the satisfaction about scar appearance was worse. There were low positive correlations between BIS and CS (*r* = 0.386, *P* < .01) and between BIS and self-confidence (*r* = 0.366, *P* < .01). This means that the higher the body image, the better the self-confidence and satisfaction of scar appearance were. There was moderate positive correlation between CS and self-confidence (*r* = 0.582, *P* < .01). This means that the higher the satisfaction of scar appearance, the better the self-confidence was.

**Table 4:** Body image questionnaire for upper midline incision in the living donors.

	Good scarring	Bad scarring	
Question	n = 34	n = 58	*P*
Body image (5)			
1. Are you less satisfied with your body since the operation?	3.44 ± 0.96	2.97 ± 1.15	.037
2. Do you think the operation has damaged your body?	2.74 ± 1.02	2.64 ± 1.12	.679
3. Do you feel less attractive as a result of your operation?	3.71 ± 0.72	3.33 ± 1.03	.042
4. Do you feel less feminine/masculine as a result of your operation?	3.62 ± 0.85	3.36 ± 0.97	.191
5. Is it difficult to look at yourself naked?	3.62 ± 0.85	3.52 ± 0.92	.606
Total	17.12 ± 3.21	15.66 ± 3.98	.057
**Cosmetic (3)**			
6. On a scale from 1 to 7, how satisfied are you with your scar?	5.47 ± 1.46	4.44 ± 1.27	<.001
7. On a scale from 1 to 7, how would you describe your scar?	5.50 ± 1.52	4.22 ± 1.39	<.001
8. Could you score your own scar on a scale from 1 to 10?	7.91 ± 2.09	5.86 ± 2.01	<.001
Total	18.88 ± 4.89	14.48 ± 4.48	<.001
**Self-confidence (2)**			
9. How confident were you before your operation?	8.12 ± 1.47	7.72 ± 1.63	.250
10. How confident were you after your operation?	7.59 ± 1.74	6.66 ± 1.73	.015
Total	15.71 ± 3.04	14.38 ± 3.07	.048

**Table 5:** Pearson product-moment correlation for VSS and BIQ of upper midline incision.

	1	2	3	4
1. VSS	–			
2. BIS	−0.159	–		
3. CS	−0.464^∗∗^	0.386^∗∗^	–	
4. Self-confidence	−0.194	0.366^∗∗^	0.582^∗∗^	–

BIS = body image scale, BIQ = body image questionnaire, CS = cosmetic scale, VSS = Vancouver scar scale. ^∗^*P* < .05, ^∗∗^*P* < .01.

## 4 Discussion

With technical advancements and more experiences, there has been a significant decrease in donor morbidity in living donor hepatectomy. However, the permanent impact of donation was scar appearance and a change in body image. In the present study, we found the upper midline incision scar was worse than the transverse incision scar (*P* < .001). Thence, the study only assessed the independent risk factors for poor upper midline scarring. The result demonstrated that male donors had a higher rate of displeasing upper midline incision scars in comparison with the female donors.

Many molecular and clinical data support that estrogen might accelerate the wound-healing process by dampening inflammatory reaction, causing rapid epithelialization, stimulating granulation formation, and enhancing matrix deposition, especially in the remodeling phase of wound healing.^[15–17]^ However, different types of skin injuries, such as burns or surgical incisions, could induce different degrees of scar formation, scar contracture, and abnormal scar progression/generation outcomes. Previous studies indicated that being female was one of the independent risk factors for postburn pathologic scarring.^[18,19,20]^ Lineal scar results in total knee replacement and total hip replacement show that the incidence rate of scarring was no different between females and males.^[21]^ A literature provided an overview of risk factors for hypertrophic scarring, which included young age, bacterial colonization, and skin subjected to stretching, but hypertrophic scarring was not associated with gender.^[22]^ This study supports the finding that female donors experience better upper midline incision scar outcomes than male donors; this may result from the beneficial effects of estrogen. At same time, poor compliance of scar caring on the part of the male donor population. Only 40% of male donors could follow the post-operative scar care protocol for 6 months in this study. It may another reason that caused the obvious scarring in male donors.

Postoperative scarring is one kind of symbol of the recovery process. Experiencing a normal healing process and having a scar in good condition can provide donors physical satisfaction.^[23]^ Living liver donors were reported to experience a negative impact on appearance satisfaction. Approximately 28% of living donors reported a surgical wound that was “worse than expected”, and one-third of all living donors answered that the surgery made a “change” in their physical appearance.^[24]^ Body image was affected in 24% of donors; they found themselves less appealing due to obvious scar formation.^[25]^ Postdonation body image and cosmetic satisfaction of scar appearance were even significantly lower for living donor hepatectomy in comparison with living donor nephrectomy.^[26]^ In our body image questionnaire result, only cosmetic satisfaction and self-confidence presented significant differences. An obvious scar leads to an inferior cosmetic result and lower self-confidence. Our two outcomes were similar to another study.^[2]^

Although the scarring brings some disadvantages in this study, most donors still expressed that they gained positive psychological outcomes. They felt they were “better persons” for having donated. Consistent with many literature, donors completely endorsed positive feelings about the donation and would make the same decision to donate again.^[27–29]^ Based on the altruistic character of donation, a donor is usually motivated by the desire to save their recipient's life.^[28]^ After donating, 14% to 31% of donors reported improved self-esteem^[27,30]^ and 65% reported feeling a general benefit.^[25]^ Therefore, the donation-related psychological benefit may be a reason why there was no significant difference between the two groups in the BIS. However, there was a positive correlation between BIS and CS and between BIS and confidence, so a donor's change in body image still deserves attention.

Scars represent the visible sequelae of surgery. Some strategies to impede the development of aesthetically unpleasant scars are scar massage, tension reduction, wound edge eversion, and the use of onion extract, silicone, or pressure garments.^[31]^ Clinically, the largest modifiable factor of scar formation is the design of the skin incision since it relates to the amount of tension in the incision during the postoperative period. One study already demonstrated that reducing skin tension is a highly effective method for scar prevention and treatment.^[32]^ Incisions made parallel to the Langer's lines may heal better and produce less scarring than those that cut across the Langer's lines.^[6,32]^ However, donors should be encouraged to take active participation in wound care, even long after the skin appears to have healed.

Our study has a number of limitations. First, this is a cross-sectional study; data collection was not very complete. Second, the BIQ was not designed to assess body image and appearance for living liver donors. It may not fully capture the subtle psychological changes in living donors.

## 5 Conclusion

The upper midline incision and male donors have higher rates of scarring in comparison with the transverse incision and female donors. Poor scarring affects the cosmetic satisfaction of living liver donors, but it does not affect body image. Even though the donor's scar had been healing, medical staff still encouraged donors to take active participation in wound care and continuously observed the impact of surgical scars on psychological changes in living liver donors.

## Author contributions

**Conceptualization:** Ya-Lan Hsu, Chia-En Hsieh, Li-Chueh Weng, Yao-Li Chen.

**Data curation:** Ping-Yi Lin, Shin-Lung Lin.

**Investigation:** Shin-Lung Lin, Kuo-Hua Lin.

**Methodology:** Li-Chueh Weng, Yao-Li Chen.

**Project administration:** Ya-Lan Hsu, Chia-En Hsieh.

**Supervision:** Li-Chueh Weng, Yao-Li Chen.

**Validation:** Li-Chueh Weng, Yao-Li Chen.

**Visualization:** Ya-Lan Hsu.

**Writing – original draft:** Ya-Lan Hsu, Chia-En Hsieh.

**Writing – review & editing:** Li-Chueh Weng, Yao-Li Chen.
